# Core-shell nanowire arrays based on ZnO and Cu_x_O for water stable photocatalysts

**DOI:** 10.1038/s41598-019-53873-0

**Published:** 2019-11-21

**Authors:** Camelia Florica, Andreea Costas, Nicoleta Preda, Mihaela Beregoi, Andrei Kuncser, Nicoleta Apostol, Cristina Popa, Gabriel Socol, Victor Diculescu, Ionut Enculescu

**Affiliations:** 1 National Institute of Materials Physics, Multifunctional Materials and Structures Laboratory, Functional Nanostructures Group, 405A Atomistilor Street, 077125 Magurele, Ilfov Romania; 2 National Institute for Laser, Plasma and Radiation Physics, 409 Atomistilor Street, 077125 Magurele, Ilfov Romania

**Keywords:** Photocatalysis, Nanowires

## Abstract

Staggered gap radial heterojunctions based on ZnO-Cu_x_O core-shell nanowires are used as water stable photocatalysts to harvest solar energy for pollutants removal. ZnO nanowires with a wurtzite crystalline structure and a band gap of approximately 3.3 eV are obtained by thermal oxidation in air. These are covered with an amorphous Cu_x_O layer having a band gap of 1.74 eV and subsequently form core-shell heterojunctions. The electrical characterization of the ZnO pristine and ZnO-Cu_x_O core-shell nanowires emphasizes the charge transfer phenomena at the junction and at the interface between the nanowires and water based solutions. The methylene blue degradation mechanism is discussed taking into consideration the dissolution of ZnO in water based solutions for ZnO nanowires and ZnO-Cu_x_O core-shell nanowires with different shell thicknesses. An optimum thickness of the Cu_x_O layer is used to obtain water stable photocatalysts, where the ZnO-Cu_x_O radial heterojunction enhances the separation and transport of the photogenerated charge carriers when irradiating with UV-light, leading to swift pollutant degradation.

## Introduction

To answer the requirement of decreasing the use of fossil fuels researchers are directing their efforts towards harvesting solar, wind, bio-mass and other forms of renewable energy^1,2^. Developing suitable and sustainable materials for applications such as solar cells, batteries, H_2_ generation and pollutants removal via photocatalysis represents a sensible approach for achieving this goal^1,3–6^. In recent years, the trend in obtaining new material architectures with improved functionalities shifted towards nanoscale engineering, the advantages include minimized substance consumption and improvement of device performances^7^. Among nanostructures, nanowires are particularly remarkable, because of their high aspect ratio and large surface area^8^ they can show enhanced optical properties such as light trapping, antireflection, and high absorption^9–11^. To be technologically feasible, nanowire fabrication must be fast, controlled and easily scalable which is achievable with appropriate synthesis routes and material combinations^12–14^. For semiconductors with low exciton and carrier diffusion lengths, free charge carriers can be generated and transferred with a higher efficiency for radial heterojunctions than for planar/normal ones^15,16^ due to the orthogonal directions of the light absorption and charge in core-shell radial heterojunctions. In this particular case, photon absorption takes place along the nanowire length, while the separation of charges takes place within the diameter. Arrays of core-shell nanowires and/or nanowire nanoparticle junctions based on Pd/ZnO, ZnO/ZnFe_2_O_4_, ZnO/Zn_2_TiO_4_, ZnO/ZnS/Au, TiO_2_/CuO, Si/Cu_2_O are reported to be used in photocatalytic applications such as water splitting and pollutants removal^3,17–22^, dissolution processes not being investigated.

As a cost-effective alternative for obtaining photocatalysts that are efficient and also water stable, in this work ZnO–Cu_x_O core-shell nanowire arrays are used as p-n staggered gap radial heterojunctions. A good photocatalytic efficiency and protecting the ZnO nanowire from dissolution are advantages of this system. Zinc oxide is an n-type semiconductor with a direct wide band-gap (E_g_ = 3.3 eV), a large exciton binding energy (~60 meV) and high electron mobility^13,23,24^. For aqueous environment applications, a major disadvantage to be overcome is the ZnO dissolution. This process was observed previously for nanoparticles^25–29^, thin films^30,31^, porous nanosheets^32^, and even nanowires^33–35^, being enhanced under UV-light irradiation^32,36^, but was not discussed, up to now, to our knowledge, in relation with its photocatalytic response. On the other hand, copper oxide is a p-type semiconductor, abundant, cheap and can be found in the form of: Cu_2_O, with a direct band-gap of 2.0 eV, CuO, with an indirect band-gap of 1.2–1.6 eV^37,38^ or a mixture between them, particular composition being a consequence of the preparation method.

In our study, ZnO nanowires were obtained by thermal oxidation in air and covered with Cu_x_O layers deposited by magnetron sputtering leading to water stable photocatalysts. Electrical charges transfer along the radial junction and between the ZnO-Cu_x_O core-shell nanowires and an aqueous media are investigated for different thickness of the shell layer in order to explain the mechanism of the photocatalytic degradation of methylene blue. Herein, for ZnO nanowires, two concurrent processes are considered: its dissolution which leads to zinc ions release in the solution and the charge carriers photogeneration. The dissolution process of ZnO nanowires with the covering by Cu_x_O decreases with increasing the Cu_x_O thickness, while the charges photogeneration improves. When reaching an optimum thickness of Cu_x_O, the staggered gap radial heterojunction based on ZnO-Cu_x_O nanowires has a better photocatalytic response than the pristine ZnO nanowires, being in the same time water stable.

## Materials and Methods

### Materials

All solvents and chemical reagents are commercially available being purchased and used without further purification.

### Preparation of core-shell nanowires arrays

The synthesis of the ZnO-CuxO core-shell nanowires was described in our previous paper^39^. Thus, ZnO nanowires were prepared using 2 cm^2^ zinc foils (Alfa Aesar, 99.99%). Initially, the metallic foils were cleaned for 5 min in an ultrasonic bath (acetone and isopropyl alcohol), followed by repeated rinsing in deionized water and dried under nitrogen gas flow. The cleaned zinc substrates were thermally oxidized in air in a convection oven at 500 °C for 12 h. The length and diameters of the ZnO nanowires are controlled by the thermal oxidation parameters (temperature and time), as reported in our previous study^23,24^. Further, the ZnO-Cu_x_O core-shell nanowires were obtained by depositing copper oxide thin layers in a TECTRA magnetron sputtering system from a CuO target (Kurt Lesker)^39^. The pressure used was 5.4 × 10^−3^ mbar in Ar atmosphere and the applied power was 100 W. By changing the deposition time from 6 min to 18 min and 30 min copper oxide thin films with different thicknesses were obtained, samples denoted with ZnO-Cu_x_O_1, ZnO-Cu_x_O_2 and ZnO-Cu_x_O_3, respectively^39^. Also, for comparison reasons, a copper oxide layer was deposited on Si/SiO_2_ substrate for 30 min (sample denoted with Cu_x_O).

**Figure 1:**
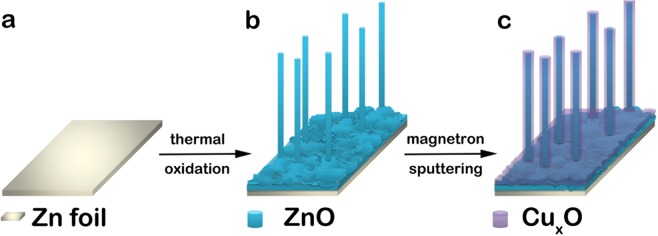
Illustration of the preparation procedures for ZnO – Cu_x_O core-shell nanowire arrays: (**a**) cleaning the Zn foil used for substrate and growth site, (**b**) synthesizing ZnO nanowires by thermal oxidation in air and (**c**) obtaining ZnO – Cu_x_O core-shell nanowire arrays by depositing a layer of Cu_x_O by magnetron sputtering.

The stages implicated in the synthesis of the ZnO-CuxO core-shell nanowires^39^ are illustrated in Fig. 1.

### Characterization

The characterization techniques used for evaluating the properties of the ZnO-CuxO core-shell nanowires were detailed in our previous paper^39^. Thus, the structural, morphological and optical properties of the thermally oxidized Zn foil and of the samples covered with copper oxide were evaluated using a Bruker AXS D8 Advance instrument with Cu K_α_ radiation (λ = 0.154 nm), a Zeiss Merlin field emission scanning electron microscope (FESEM), a Perkin–Elmer Lambda 45 UV–vis spectrophotometer equipped with an integrating sphere and a FL 920 Edinburgh Instruments spectrometer with a 450 W Xe lamp excitation and double monochromators, respectively^39^. In the X-ray diffraction (XRD) measurements, the source was operated at 40 kV and 40 mA eliminating K_β_ radiation with a nickel filter. Also, transmission electron microscopy (TEM) investigations using several techniques were performed on a Cs probe corrected JEM ARM 200F microscope provided with a JEOL energy-dispersive X-ray spectroscopy (EDS). Scanning transmission electron microscopy (STEM) was combined with the EDS analytical technique in order to map the local chemical composition. For TEM analysis, the ZnO and ZnO-Cu_x_O nanowires were dispersed into isopropyl alcohol by sonication and in order to be investigated a drop of solution containing nanowires was placed on the TEM grid. The chemical analysis was obtained by means of EDX^39^.

X-Ray Photoelectron Spectroscopy (XPS) has been performed in an AXIS Ultra DLD (Kratos Surface Analysis) setup equipped with an 180° hemispherical analyzer^39^, using Al K_α1_ (1486.74 eV) radiation produced by a monochromatized X-Ray source at operating power of 300 W (12 kV × 25 mA). The base pressure in the analysis chamber was at least 1.0 × 10^−8^ mbar. Partially charge compensation was reached by using a flood gun operating at 1.52 A filament current, 2.73 V charge balance, 2.02 V filament bias. The survey spectra have been recording using hybrid lens mode, 80 eV pass energy, slot aperture. High resolution core level spectra have been recorded using Field of View 2 lens mode, 20 eV pass energy, 110 µm aperture. The binding energy scale was calibrated to the C 1 s standard value of 284.6 eV.

The electrochemical studies were carried out using a VoltaLab PGZ100 potentiostat, running VoltaMaster 4.0 software. The experiments were done in a three-electrodes configuration, which consisted of a saturated calomel electrode (SCE) as reference, a platinum wire as auxiliary and the ZnO nanowires or ZnO-Cu_x_O core-shell nanowires as working electrode. The electrochemical impedance spectroscopy (EIS) measurements were made in 0.1 M KCl, at open circuit potential (OCP) values, using a perturbation of 10 mV for 60 harmonic frequencies ranging from 100 kHz to 0.1 Hz at 10 steps/decade. The impedance spectra were analysed by fitting with an equivalent electrical circuit using ZView software (Scribner Associates, USA)^40^ containing as parameters *R*_*s*_, *CPE* and *W*_*s*_. The *R*_s_ consists of the solution and the bulk composite resistances. The constant phase elements, defined as:

a $$CPE=-\,{(Ci\omega )}^{-\alpha }$$

is modelled as a non-ideal capacitor where the capacitance *C* describes the charge separation at the double layer interface and the *α* exponent is due to the heterogeneity of the surface.

The definition of the Warburg element used is:

b $${W}_{s}={R}_{{\rm{diff}}}{(it\omega )}^{-{\rm{n}}}\,\tanh ({[it\omega ]}^{{\rm{n}}})$$

where *R*_diff_ is a diffusion resistance of electroactive species, *τ* is a time constant depending on the diffusion rate (*τ* = *l*^2^/*D*, where *l* is the effective diffusion thickness, and *D* is the effective diffusion coefficient of the species), and *n* = 0.50 for a perfect uniform flat interface^41^.

The photocatalytic activity of the synthesized samples under UV irradiation was evaluated by measuring the optical absorbance of the methylene blue (MB) solution at 665 nm wavelength using an UV–visible spectrophotometer (Evolution 220, Thermo Scientific). In this respect, a 15 watts UV bench lamp that emitted at wavelength 312 nm was used as a light source. The tests were carried out in a flask type reactor of glass with 10 mL of methylene blue aqueous solution adjusted to be 35 μM and pH of 7 where the zinc foils containing arrays of nanowires were immersed. The dye degradation was performed under constant stirring, in dark and UV conditions. After every 20 min of UV irradiation, the solution was withdrawn in order to collect its optical absorbance spectrum. During the UV irradiation, the MB solution was kept at 24 °C by means of circulating bath model TC120 with refrigerator from Grant. To make a comparison of the photocatalytic activity of the investigated samples, the degradation efficiency was estimated using the equation:

c $$Degradation\,efficiency=({C}_{0}-C)/{C}_{0}\times {100}$$

where, *C*_0_ is the initial value of the dye concentration, *C* is the value of dye concentration at *t*, time. Also, the degradation rate constant was obtained from the slope of the linear fitting of *ln(C*_0_*/C) vs. time*, taking into consideration that the photocatalytic degradation of MB is classified as the first-order Langmuir–Hinshelwood kinetics described by the equation:

d $$ln({C}_{0}/C)=kt$$

where *C*_0_ is the initial concentration of MB, *C* is the concentration of MB at a time *t*, and *k* is the first-order degradation rate constant.

## Results and Discussion

### Morphological, structural, compositional and optical properties

**Figure 2:**
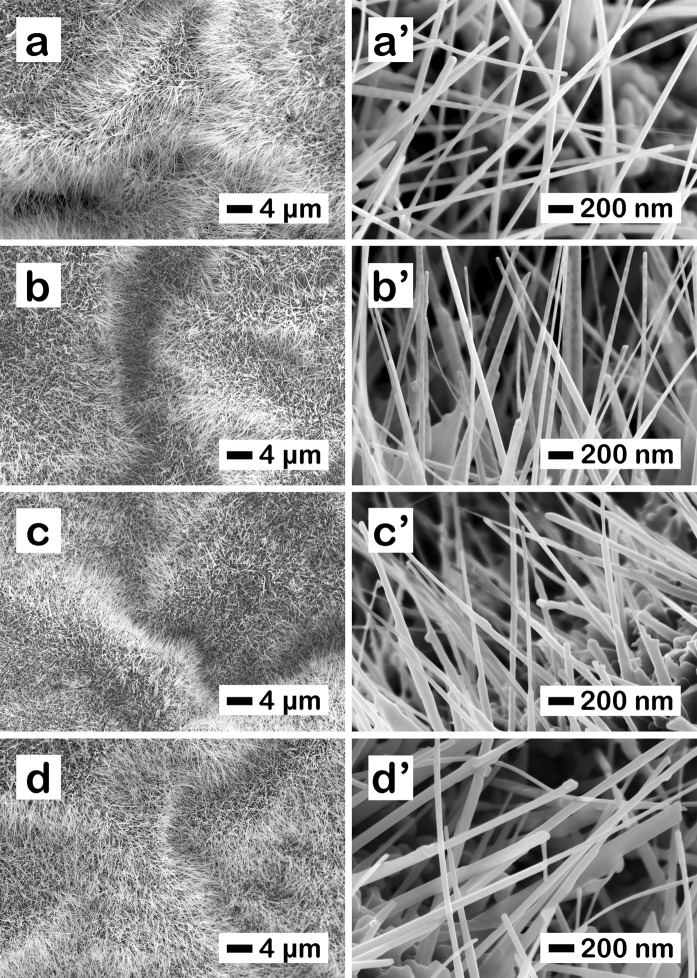
FESEM images at different magnifications for the as prepared (**a,a’**) ZnO nanowires, (**b,b’**) ZnO – Cu_x_O_1, (**c,c’**) ZnO – Cu_x_O_2 and (**d,d’**) ZnO – Cu_x_O_3 core-shell nanowires.

The morphology investigation revealed that the ZnO nanowires have a high density onto the Zn foil, Fig. 2, with lengths up to 30 µm and diameters of about 30 nm, Fig. 2(a,a’). The deposition of the Cu_x_O layer led to an increase in the diameter of the ZnO-Cu_x_O nanostructure. Consequently, at 6 min (Fig. 2(b,b’)), 18 min (Fig. 2(c,c’)) and 30 min (Fig. 2(d,d’)) deposition time, the thickness of the nanowire increased, in average with about 10 nm for ZnO-Cu_x_O_1, 20 nm for ZnO-Cu_x_O_2 and 30 nm for ZnO-Cu_x_O_3, respectively. Thus, the increase in the diameter of the ZnO nanowires after their coating with the Cu_x_O layer suggests the formation of a heterojunction between the two semiconductors.

**Figure 3:**
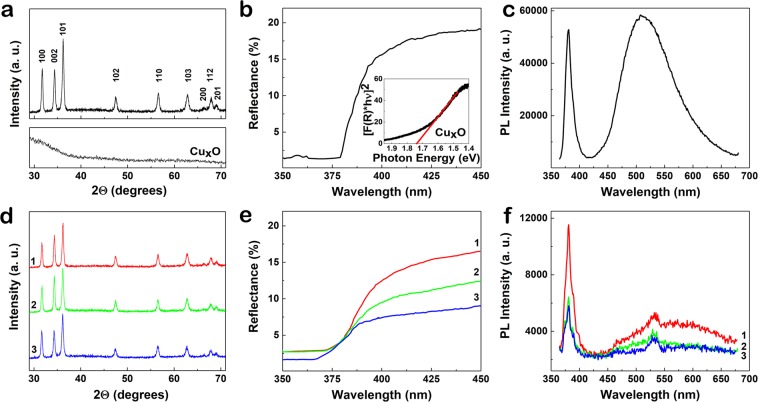
(**a,d**) XRD, (**b,e**) reflectance and (**c,f)** photoluminescence spectra of (a – up, **b,c**) ZnO and (**d–f)** ZnO-Cu_x_O core shell nanowires: ZnO-Cu_x_O_1 (red curves), ZnO-Cu_x_O_2 (green curves) and ZnO-Cu_x_O_3 (blue curves) nanowires; (a–down) XRD and (b – inset) Kubelka-Munk representation of the Cu_x_O layer with the longest deposition time.

The XRD analysis showed the hexagonal wurtzite crystalline structure of the ZnO nanowires, Fig. 3(a) up, where the Miller indexes are depicted for every peak: (100), (002), (101), (102), (110), (103), (200), (112) and (201) corresponding to JCPDS file no. 36–1451. The same reflecting planes were also identified in the XRD patterns of ZnO-Cu_x_O_1, ZnO-Cu_x_O_2 and ZnO-Cu_x_O_3 core- shell nanowire samples, Fig. 3(d). It has to be noticed that in the diffractograms of the core-shell nanowire arrays there is no additional peak that can be related with the Cu_x_O layer. The result can be explained taking into account the amorphous nature of the copper oxide films deposited by radio-frequency magnetron sputtering^42,43^ evidenced by the XRD pattern of the Cu_x_O film deposited on Si/SiO_2_ substrate (Fig. 3(a) down) in the same condition with that obtained in the case of ZnO-Cu_x_O_3 sample.

The reflectance measurements revealed that the energy band gap of ZnO does not alter significantly with the deposition of the Cu_x_O shell layers being around 3.3 eV (Fig. 3(b,e)). The energy band gap of the Cu_x_O on SiO_2_/Si was estimated from the Kubelka-Munk representation to be 1.74 eV (inset of Fig. 3(b)), a value between the ones of CuO and Cu_2_O nanostructures^37,38^. A decrease in reflectance in the visible range with the increase in thickness of Cu_x_O layer is noticed, going from 19% for ZnO nanowires down to 17% for ZnO-Cu_x_O_1, 12.5% for ZnO-Cu_x_O_2 and 7.5% for ZnO-Cu_x_O_3 core-shell nanowires. Thus, the ZnO nanowires covered with the thickest Cu_x_O layer (15 nm) give rise to an increase of about 40% in the absorbance of the resulting ZnO-Cu_x_O core-shell nanowires in the visible part of the solar spectrum, useful for photocatalysis applications.

For ZnO nanowires, the photoluminescence spectrum (Fig. 3(c)) exhibits a typical response for this semiconductor when being excited with 350 nm. The sharp peak at 380 nm, corresponds to the band-to-band transitions in ZnO (excitonic peak), while the broad intense emission band in the visible region, attributed to optical active defects in ZnO^24,25^. A decrease in the intensities of the two emission bands takes place when the ZnO nanowires are covered with Cu_x_O layers. Also, a change in the ratio of the emission bands is observed, Fig. 3(f). This effect is in agreement with the compensation of ZnO surface defects by the covering with Cu_x_O layers, being probably due to decrease of scattering processes^44^.

**Figure 4:**
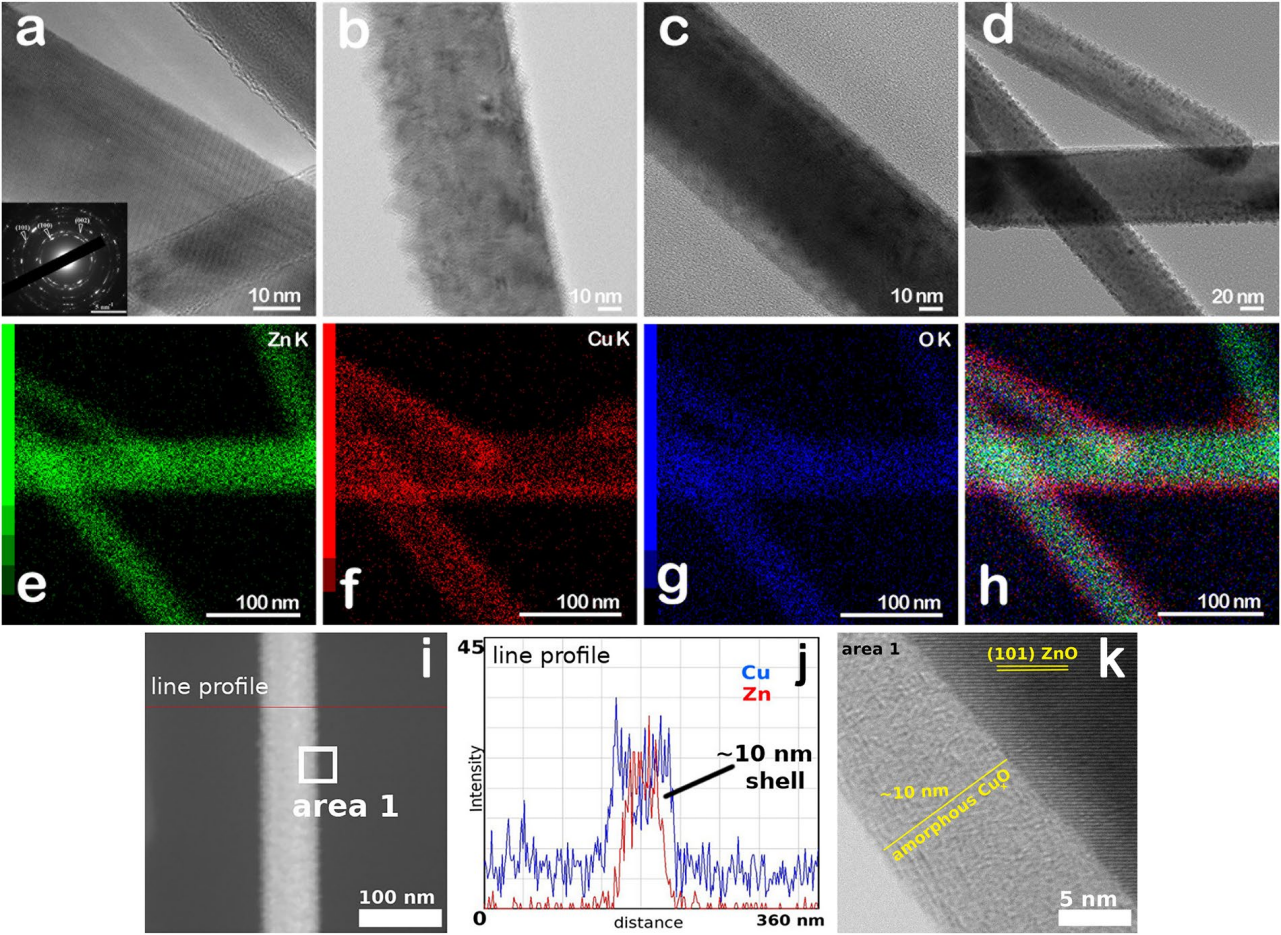
TEM images of (**a**) ZnO nanowires having in the inset a SAED pattern proving the ZnO wurtzite phase and of (b-d) ZnO-Cu_x_O core-shell nanowires with different shell thicknesses (with (**b**) ZnO-Cu_x_O_1, (**c**) ZnO-Cu_x_O_2 and (**d**) ZnO-Cu_x_O_3), (**e–h**) Elemental maps of the same ZnO-Cu_x_O_3 nanowires region, indicating spatially-resolved elemental distribution of Zn (green), Cu (red) O (blue), and their superposition, (**i**) STEM image, (**j**) EDS line profile analysis by STEM mode and (**k**) HRTEM image of area 1 of a ZnO-Cu_x_O core-shell nanowire.

In Fig. 4(a–d), the TEM images show ZnO nanowires and ZnO-Cu_x_O core-shell nanowires proving the difference in the shell thicknesses with increasing the deposition time, in accordance with the FESEM observations. Thus, the ZnO nanowires have diameters of about 30 nm while the shell thicknesses for the core-shell nanowires presents average values of about 5 nm for ZnO-Cu_x_O_1, 10 nm for ZnO-Cu_x_O_2 and 15 nm for ZnO-Cu_x_O_3. For ZnO nanowires, the SAED pattern evidenced the wurtzite phase (inset of Fig. 4(a)) with the (101), (100) and (002) planes, consistent with the XRD analysis, while the Cu_x_O layers were amorphous (Fig. 4(b–d)). Furthermore, the TEM image in Fig. 4(b) reveals that the surface of the ZnO-Cu_x_O_1 core-shell nanowire is relative rough, probably due to discontinuities in the Cu_x_O thin film, which does not entirely cover the ZnO nanowire surface. With increasing the Cu_x_O thin film thickness, the surface of the core-shell nanowires becomes smoother, decreasing the number of possible pinholes in the Cu_x_O layer and consequently leading to a complete coverage of the ZnO core for the samples denoted by ZnO-Cu_x_O_3 (Fig. 4(d)). The elemental maps displayed in Fig. 4(e–h) demonstrate the spatial distribution of all constituting elements for the sample with the highest Cu_x_O thickness, ZnO-Cu_x_O_3. The Zn K-edge signals are emitted from the core area, while the Cu K-edge is seen at the edges, having O K-edge uniformly distributed along the nanowire, proving the heterojunction between ZnO and Cu_x_O in the core-shell nanowire. The EDS line profile analysis by STEM mode and the HRTEM image (Fig. 4(i–k)) reveal also the formation of a core-shell structure in the nanowires consisting in a hexagonal wurtzite ZnO core and an amorphous Cu_x_O shell. Additionally, TEM analysis confirm the diameters of the core-shell nanostructures determined by FESEM. Thus, the ZnO nanowires have diameters of about 30 nm while the shell thicknesses for the core-shell nanowires presents average values of about 5 nm for ZnO-Cu_x_O_1, 10 nm for ZnO-Cu_x_O_2 and 15 nm for ZnO-Cu_x_O_3.

**Figure 5:**
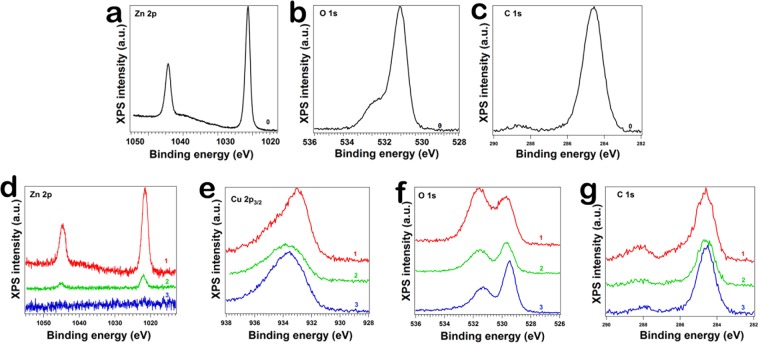
XPS spectra of the (**a,d**) Zn 2p levels, (**b,f**) O 1 s levels, (**c,g**) C 1 s levels and (**e**) Cu 2p_3/2_ levels, for pristine ZnO nanowires (**a–c**) and for ZnO-Cu_x_O core-shell nanowires (**d–g**): ZnO-Cu_x_O_1, ZnO-Cu_x_O_2 and ZnO-Cu_x_O_3 respectively.

The XPS investigation was carried out to evidence the nature of bondings and to discover the Cu oxidation state in the Cu_x_O layers. The core level spectra (Fig. 5) have been deconvoluted using Voigt profiles, based on the methods described in reference^45^ and are presented in Fig. S1 for Zn 2p levels, Fig. S2 for O 1 s levels and Fig. S3 for Cu 2p_3/2_ levels. The atomic composition, Table S1, has been determined by using the integral areas provided by the deconvolution procedure normalized at the atomic sensitivity factors^46^, taking into consideration a slight contamination of the surfaces with CO_3_.

The formation of ZnO is confirmed by XPS for the samples containing ZnO, ZnO-Cu_x_O_1, ZnO-Cu_x_O_2, with the signal fading when increasing the thickness of the Cu_x_O layer. The XPS analysis has shown that the Cu_x_O shell comprises a mixture between Cu_2_O and CuO having a ratio of about 3:1 for ZnO-Cu_x_O_1 and a ratio of 1:1 for ZnO-Cu_x_O_2 and ZnO-Cu_x_O_3. Thus, the XPS results attested also the formation of a heterojunction between ZnO and Cu_x_O in the core-shell nanowire.

### Electrochemical properties

**Figure 6:**
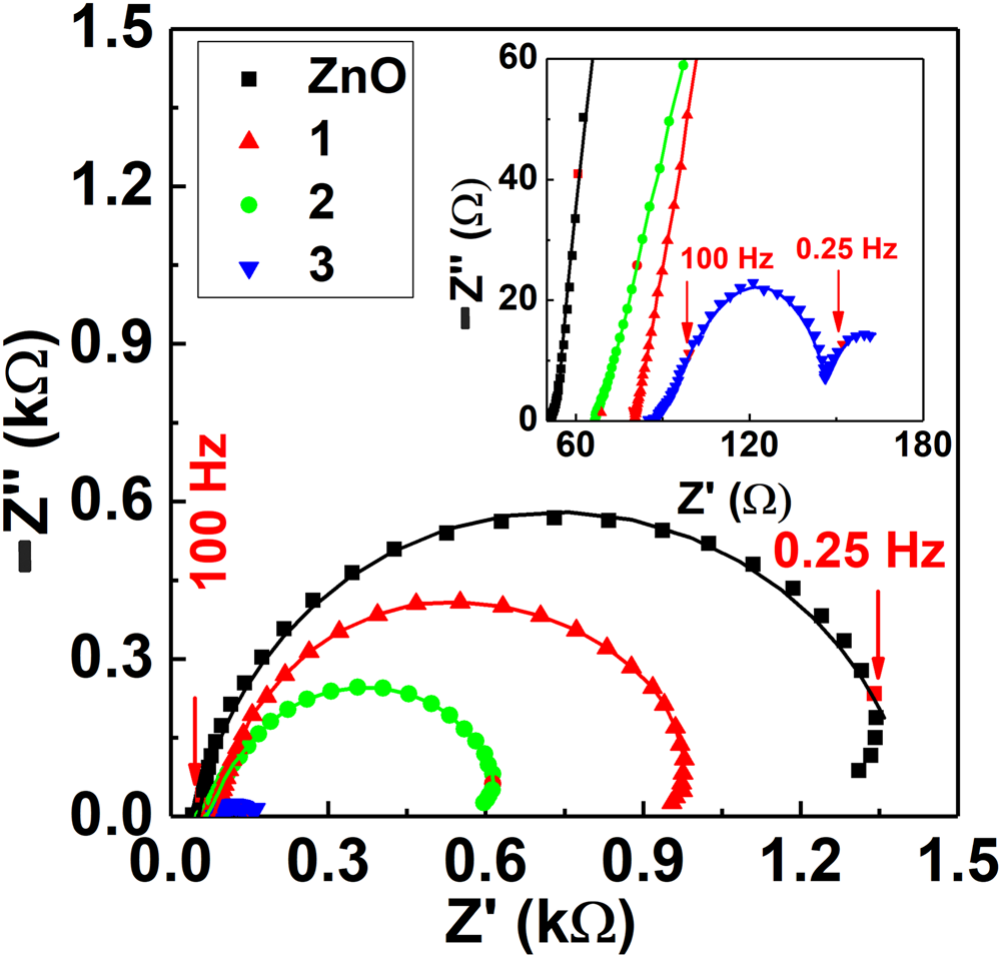
Nyquist representation of the EIS of ZnO nanowires (black squares) and of ZnO-Cu_x_O core-shell nanowires: ZnO-Cu_x_O_1 (red upward triangles), ZnO-Cu_x_O_2 (green circles) and ZnO-Cu_x_O_3 (blue downward triangles) recorded in 0.1 M KCl at OCP. The symbols represent the experimental data and the continuous lines the fitting results with the circuit in Fig. 7.

EIS was employed to investigate the electrical properties of the ZnO nanowires and ZnO-Cu_x_O core-shell nanowires and to analyse the electron charge transfer between these nanowire arrays and water based solutions. EIS was carried out in 0.1 M KCl at OCP values, Fig. 6. The OCP values (−1.015 V for ZnO, −1.020 V for ZnO-Cu_x_O_1, −1.025 V for ZnO-Cu_x_O_2 and −1.020 V for ZnO-Cu_x_O_3) were also recorded in 0.1 M KCl until a drift below 0.1 mV min^−1^ was reached. The obtained EIS included three main regions. The first region between 100 kHz and 100 Hz corresponding to the electron transfer and diffusion process. The second region between 100 Hz and 0.25 Hz, a semi-circular part due to pure electron transfer. The third region related to the frequency range below 0.25 Hz represented by an inverse loop is due to an inductive component. Thus, all characterized samples are eligible for charge transfer processes^47^.

**Figure 7:**
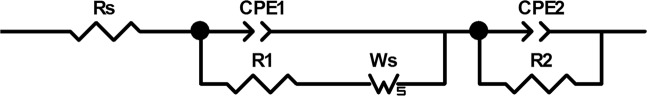
Equivalent electrical circuit used for fitting the EIS experimental data.

In agreement, the spectra were fitted with an equivalent electrical circuit, Fig. 7, formed by *R*_s_ attributed to electrochemical cell resistance, a constant phase element *CPE*_1_ in parallel with a resistor *R*_1_ and a Warburg impedance *W*_s_ corresponding to the electrolyte/electrode interface, followed by a parallel combination of a constant phase element *CPE*_2_ and a resistor *R*_2_ associated with ZnO-Cu_x_O heterojunction.

The EIS experiments revealed that, increasing the thickness of Cu_x_O layer both real and imaginary impedance decreased. Data from analysis of EIS, Table 1, showed the cell resistance *R*_Ω_ with values between 50 and 90 Ω increasing with Cu_x_O thickness. On the other hand, the charge transfer resistances *R*_1_ and *R*_2_ decreased upon increasing the thickness of the Cu_x_O, which can be associated with the protection of ZnO from dissolution, in agreement with CV studies.

**Table 1:** Values of electrical equivalent circuit elements after fitting the experimental data in Fig. 6 with the equivalent circuit in Fig. 7.

Sample	*R*_s_ (Ω)	*R*_1_ (Ω)	*CPE*_1_		*R*_2_ (Ω)	*CPE*_2_		*W*_s_		
			*C*_1_ (µF)	*α*_1_		*C*_2_ (µF)	*α*_2_	*R*_diff_ (Ω)	*Τ* (ms)	*N*
ZnO	50.8	964.6	173.9	0.87	459.6	90.3	0.99	4.7	0.6	0.32
ZnO-Cu_x_O_1	77.9	581.6	152.7	0.88	345.9	114.2	0.99	19.6	6.7	0.33
ZnO-Cu_x_O_2	65.1	319.0	231.4	0.88	227.1	378.6	0.99	27.9	11.9	0.35
ZnO-Cu_x_O_3	87.3	32.4	463.5	0.99	30.9	413.9	1.0	29.1	14.8	0.37

Also, the interfacial capacitance *C*_1_ as well as the *C*_2_ of the ZnO-Cu_x_O heterojunction showed higher values for thicker Cu_x_O layer due to an increase of the electroactive surface area, whereas the heterogeneity parameter *α*_1_ reaches higher values concurring with smother surfaces for thicker Cu_x_O. The roughness parameter *α*_2_ is approximatively 1 and remains constant, meaning that the ZnO-Cu_x_O junction behaves as a pure capacitor. Nevertheless, the diffusion resistance as well as the diffusion process time constant increased with Cu_x_O thickness, showing that when it reaches the maximum value, the faster interfacial charge transfer is attained for ZnO-Cu_x_O_3.

### Photocatalytic properties and dissolution effects

**Figure 8:**
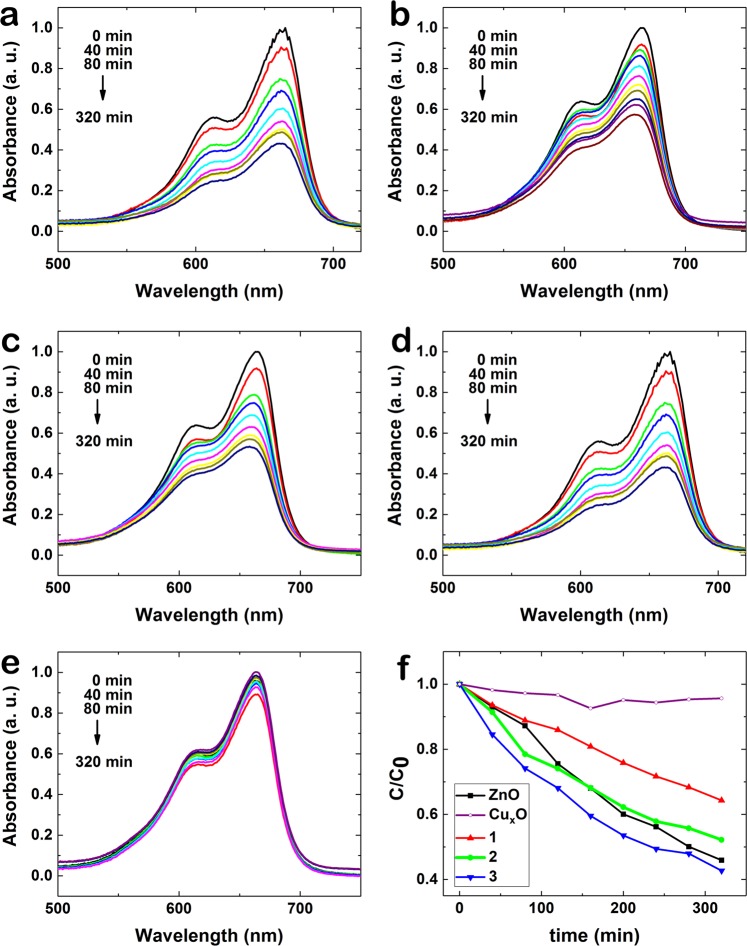
Absorption spectra showing time evolution of the degradation of MB in aqueous based solution under UV irradiation in the presence of (**a**) ZnO, (**b**) ZnO-Cu_x_O_1, (**c**) ZnO-Cu_x_O_2, (**d**) ZnO-Cu_x_O_3 nanowires (**e**) Cu_x_O film and (**f**) degradation profiles of MB in aqueous based solution under UV irradiation over time for all samples.

In order to demonstrate the photocatalytic efficiency of ZnO-Cu_x_O nanostructures, the photodegradation of the model dye methylene blue (MB) was investigated and can be observed in Fig. 8, where the peaks of absorbance curves corresponding to MB decrease in time in the presence of ZnO (Fig. 8(a)), ZnO-Cu_x_O_1 (Fig. 8(b)), ZnO-Cu_x_O_2 (Fig. 8(c)), ZnO-Cu_x_O_3 (Fig. 8(d)) and Cu_x_O (Fig. 8(e)). The degradation profiles of MB in aqueous based solution under UV irradiation over time (Fig. 8(f)) show less steep curves of ZnO-Cu_x_O_1 and ZnO-Cu_x_O_2 than pristine ZnO nanowires.

**Figure 9:**
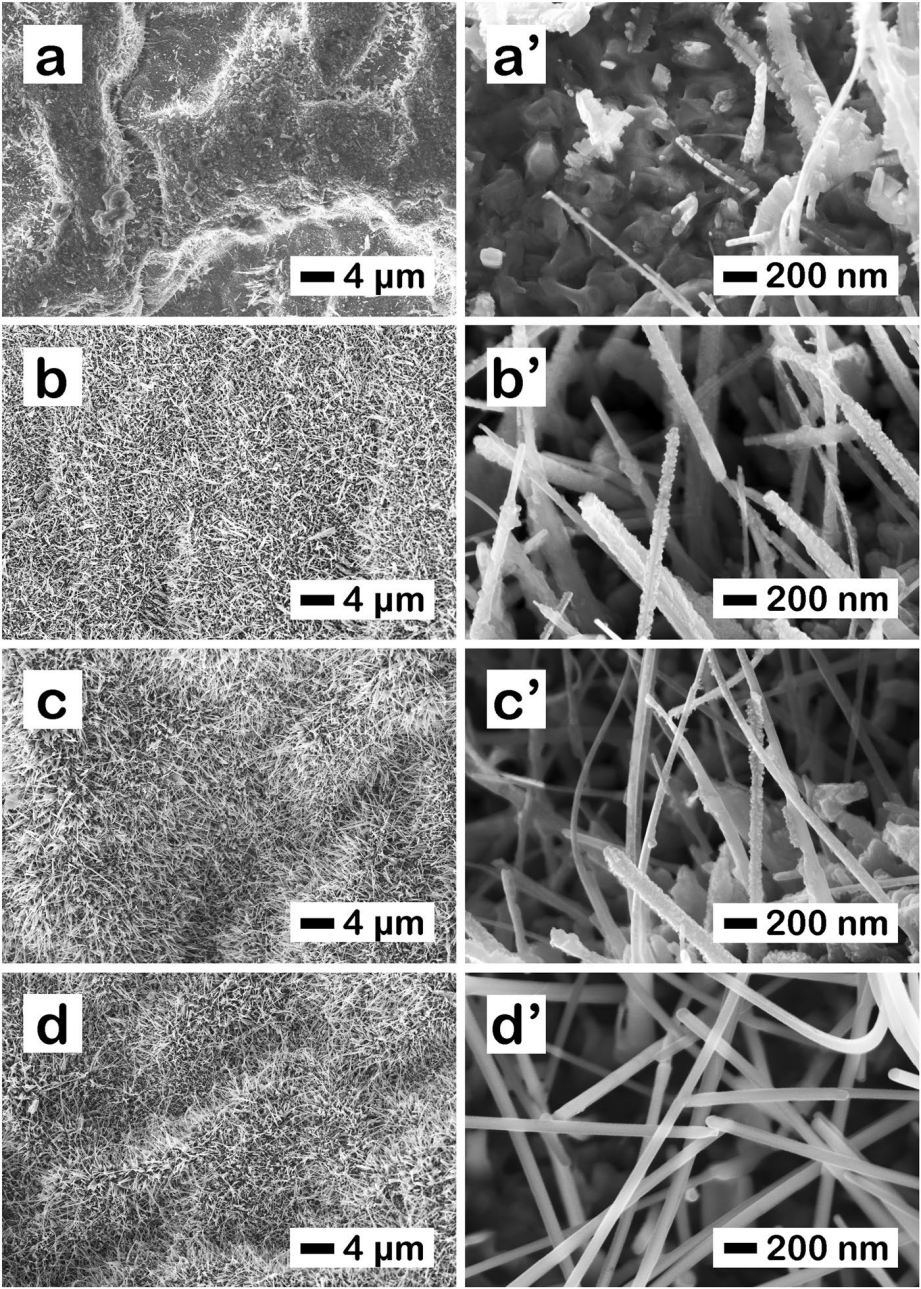
FESEM images at different magnifications for (**a,a’**) ZnO, (**b,b’**) ZnO-Cu_x_O_1, (**c,c’**) ZnO-Cu_x_O_2 and (**d,d’**) ZnO-Cu_x_O_3 nanowires after the photocatalysis experiments.

For a better understanding of the photocatalytic activity of our samples, FESEM images at different magnifications were made after the photocatalysis experiments (after immersion in water based solution and UV irradiation for at least 5 h, Fig. 9). The FESEM images revealed increased stability of the nanowires in aqueous based solutions with increasing shell thickness in agreement with the EIS results. ZnO pristine nanowires dissolve completely in 5 h (Fig. 9(a,a’)), ZnO-Cu_x_O_1 and ZnO-Cu_x_O_2 partially dissolve (Fig. 9(b,b’,c,c’**)**, respectively) in 5 h, while ZnO-Cu_x_O_3 nanowires remain morphologically unchanged (Fig. 9(d,d’)) after the photocatalysis experiments.

**Figure 10:**
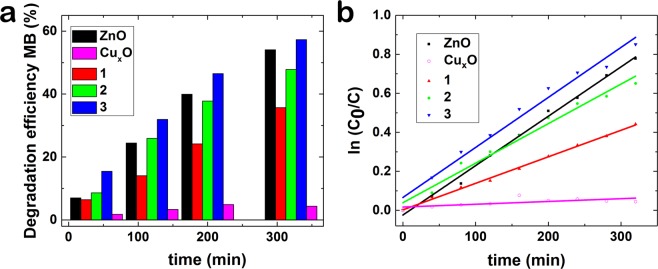
(**a**) Photocatalysis degradation efficiency of MB over time and (**b**) kinetic curves for photocatalytic degradation of MB, under UV irradiation and in the presence of ZnO, ZnO-Cu_x_O_1, ZnO-Cu_x_O_2, ZnO-Cu_x_O_3 nanowire arrays and Cu_x_O film.

**Figure 11:**
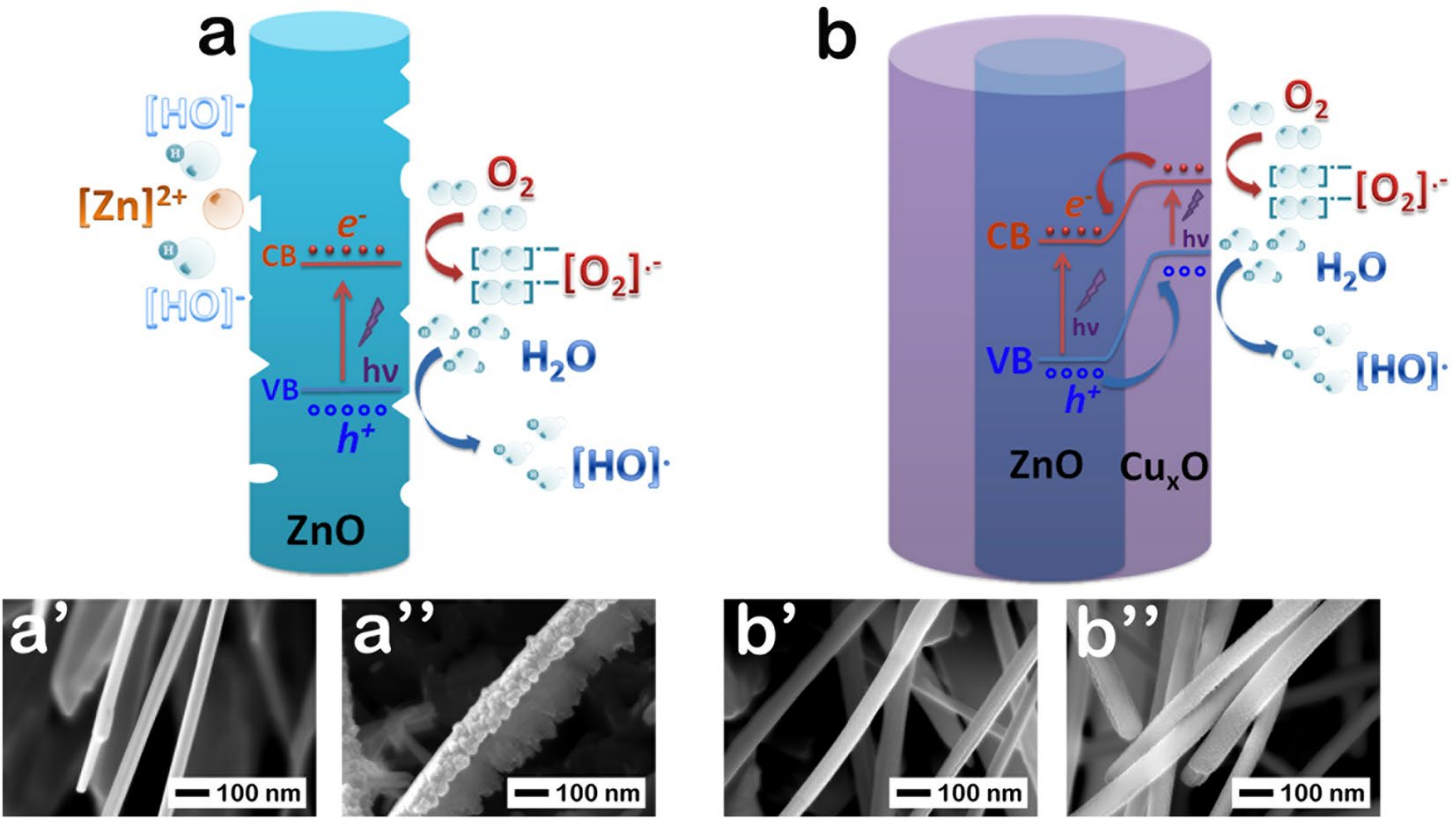
The MB photocatalysis degradation mechanism under UV irradiation in the presence of (**a**) ZnO and (**b**) ZnO-Cu_x_O_3 nanowires and the FESEM images of the samples (**a’,b’**) before and (**a”,b”**) after the photocatalysis experiment in each case, showing the (**a’**,**a”**) ZnO instability and (**b’,b”**) ZnO-Cu_x_O_3 stability in aqueous based solutions.

The degradation efficiency over time and the kinetics of the degradation of MB (*ln(C*_0_*/C) vs. time*) in the presence of ZnO, Cu_x_O, ZnO-Cu_x_O_1, ZnO-Cu_x_O_2 and ZnO-Cu_x_O_3 samples are shown in Fig. 10(a,b). An illustration of the mechanisms involved in the photodegradation of MB using ZnO and ZnO-Cu_x_O nanowires is depicted in Fig. 11.

The degradation of MB by ZnO occurs with 54% efficiency and 0.1518 h^−1^ degradation rate constant and follows distinct concurrent processes (dissolution of ZnO and photogeneration of charges) occurring either in the absence or under UV-light irradiation, in aqueous based solutions. The FESEM images taken before (Fig. 11(a’)) and after (Fig. 11(a”)) the photocatalysis experiments confirm the dissolution of ZnO nanowires in water based solution. The lower photocatalytic activity of the Cu_x_O thin film (lower efficiency, 4%, and degradation rate constant, k = 0.0085 h^−1^) compared with the ZnO nanowires can be given by the thickness of the layer and its 2D morphology which lead to the absorption of a low number of photons giving rise to a small number of photogenerated charges and thus *e*^*−*^ and *h*^+^ on its surface. For planar structures, the photogenerated carrier number increases with the thicknesses of the films^48–51^.

The ZnO-Cu_x_O_1 and ZnO-Cu_x_O_2 nanowires degrade MB with a higher efficiency (35% and 47% respectively) and a higher degradation rate constant (k = 0.0816 h^−1^ and k = 0.1218 h^−1^) than Cu_x_O, even though lower than that of pristine ZnO nanowires. For these 2 types of samples the Cu_x_O layer is not thick enough for totally blocking ZnO dissolution, the water based solution being able to protrude and reach the ZnO nanowire core, dissolving it. This determines the formation of a rough interface between the ZnO and Cu_x_O layers which enhance the recombination of photogenerated charges hindering the migration of free charges towards the surface and thus, explaining the lower MB degradation efficiencies.

The ZnO-Cu_x_O_3 core-shell nanowires exhibit a slightly higher photocatalytic activity than pristine ZnO nanowire array (57% degradation efficiency and k = 0.1542 h^−1^), having the advantage of being a water stable catalyst. When the Cu_x_O layer reaches an optimum thickness, it plays a double role, on one side protecting the ZnO nanowires from corrosion and on the other side forming a ZnO-Cu_x_O core-shell radial staggered gap heterojunction which can promote charge separation for photocatalysis applications (Fig. 11(b)). The FESEM images are proving the unchanged morphology of the ZnO-Cu_x_O_3 core-shell nanowires by showing their surface before (Fig. 11(b’)) and after (Fig. 11(b”)) the photocatalysis experiments.

Taking into account that only a few studies were focused on the photocatalytic response of arrays formed by a mixture of ZnO and CuO nanowires^52^ or of CuO core - ZnO shell nanowire arrays^53,54^ and not on ZnO core - CuO shell nanowire arrays as in our study, it is difficult to compare our photocatalytic results with the data reported in the literature. Furthermore, these reports do not take into account the ZnO dissolution, process which can enhance the photocatalytic performance of their nanowires.

In core-shell radial junctions, the light absorption and charge separation directions are orthogonal, with absorption prevailing along the nanowire length (absorption depth is larger than nanowire diameter), while the separation of charges taking place within the diameter (diffusion lengths around nanowire diameters) more efficiently than in planar junctions^16^. Furthermore, when a staggered gap heterojunction (type II) is created, efficient charge separation occurs due the built in internal field formed at the interface between the two semiconductors^51^.

When irradiating the ZnO-Cu_x_O core-shell nanowires with UV light, both component semiconductors are absorbing photons and generating charges (Fig. 11(b)). ZnO has a wide band gap of about 3.3 eV, absorbing UV light and Cu_x_O has a narrow band gap of about 1.74 eV absorbing both UV and visible light. For the ZnO-Cu_x_O_3 samples, highest absorption takes place, as confirmed by the reflectance measurements. Due to the staggered gap heterojunction (Fig. 11(b)), electrons are moving from the conduction band of Cu_x_O into the conduction band of ZnO and holes are moving from the valence band of ZnO towards the valence band of Cu_x_O, thus the photogenerated charges are separated efficiently^51,55,56^. The transferred charges at the surface of the radial p-n heterojunction can be trapped by adsorbed water or oxygen molecules to generate superoxide anion radicals or hydroxyl ions (Fig. 11(b)), similar to the case of ZnO (Fig. 11(a)), leading to the degradation of MB into harmless compounds^55,56^.

As previously reported, ZnO dissolution in aqueous environment has been observed for different morphologies such as: nanoparticles^25–29^, thin films^30,31^, porous nanosheets^32^, and even nanowires^33–35^. For nanoscale ZnO the surface to volume ratio is high and pinholes or defect sites represent the starting points in the dissolution process. In the case of Cu_x_O and ZnO-Cu_x_O at highest thickness the degradation of MB takes places because of the photogeneration of charges due to irradiation.

The proposed mechanism for the degradation of MB by ZnO, Cu_x_O and ZnO-Cu_x_O_3 in the absence and under UV-light irradiation is described as follows:

(i) In the ***absence of UV-light irradiation***, dissolution of ZnO nanowires can be related to the formation of a hydroxide layer on its surface^25–30^:
  1 $$ZnO+{H}_{2}O\rightleftharpoons Zn{(OH)}_{2}$$
  2 $$Zn{(OH)}_{2}\rightleftharpoons Zn{(OH)}^{+}+H{O}^{-}$$
  3 $$Zn{(OH)}^{+}\rightleftharpoons Z{n}^{2+}+H{O}^{-}$$
  4 $$ZnO+{H}_{2}O\rightleftharpoons Z{n}^{2+}+2H{O}^{-}$$
(ii) ***Under UV-light irradiation***, the dissolution of ZnO can be enhanced^32,36^ which leads to the appearance of photogenerated charges that can be separated and sent into the conduction and valence band respectively, giving rise to even more reactive surface products which can photocorrode ZnO:

5 $$ZnO+h\nu \to {e}^{-}+{h}^{+}$$

6 $$ZnO+2{h}^{+}\to Z{n}^{2+}+1/2\,{O}_{2}$$

7 $$ZnO+2{e}^{-}\to Z{n}^{2+}+2{O}^{-}$$

Zn^2+^ can contribute to the degradation of organic pollutants^57–59^ if, when at the surface, trap holes forming Zn^3+^ which further react with OH^−^ to produce hydroxyl radicals or capture electrons producing Zn^+^ that react with adsorbed O_2_ resulting in superoxide anion radicals as described below:

8 $$Z{n}^{2+}+{h}^{+}\to Z{n}^{3+}$$

9 $$Z{n}^{3+}+H{O}^{-}\to Z{n}^{2+}+H{O}^{\bullet }$$

10 $$Z{n}^{3+}+{e}^{-}\to Z{n}^{2+}$$

11 $$Z{n}^{2+}+{e}^{-}\to Z{n}^{+}$$

12 $$Z{n}^{+}+{O}_{2}\to Z{n}^{2+}+{{O}_{2}}^{\bullet -}$$

13 $$Z{n}^{+}+{h}^{+}\to Z{n}^{2+}$$

***Under UV-light irradiation*** at energies higher than the band gap, the electrons from the

valence band are excited and migrate towards the conduction band leaving behind an equal number of holes in the valence band. Cu_x_O and ZnO-Cu_x_O_3 are also photogenerating charges, similar to ZnO, which further can lead to the degradation of MB:

14 $$C{u}_{x}O+h\nu \to {e}^{-}+{h}^{+}$$

15 $$ZnO-C{u}_{x}O+h\nu \to ZnO({e}^{-}+{h}^{+})+C{u}_{x}O({e}^{-}+{h}^{+})$$

Some of these photogenerated charges recombine, but others travel towards the surface where they interact with adsorbed species such as *O*_2_ and *H*_2_*O* forming superoxide anion radicals and hydroxyl ions. These can form hydroxyl radicals when interacting with a hole or by subsequent reactions which lead to hydrogen peroxide that further decomposes to hydroxyl radicals. Additionally, the photogenerated holes can directly oxidize the dye molecules to organic radicals. Also, aerial oxygen acts as an electron scavenger to oxidize the activated organic. Thus, MB molecules can be photocatalitically degraded by reactive oxygen species into smaller hydrocarbons and finally into CO_2_ and H_2_O molecules^60–63^. The relevant redox reactions involved in the formation of active radicals which are responsible for MB degradation, valid also for other semiconductors used as photocatalysts (CuO, TiO_2_, ZnO-CuO as composites for core-shell nanostructures) after the photogeneration processes are summarized below^36,55,56,60–67^:

16 $${H}_{2}O+{h}^{+}\to H{O}^{-}+{H}^{+}$$

17 $$H{O}^{-}+{h}^{+}\to H{O}^{\bullet }$$

18 $${O}_{2}+{e}^{-}\to {{O}_{2}}^{\bullet -}$$

19 $${{O}_{2}}^{\bullet -}+{H}^{+}\to H{{O}_{2}}^{\bullet }$$

20 $$H{{O}_{2}}^{\bullet }+H{{O}_{2}}^{\bullet }\to {H}_{2}{O}_{2}+{O}_{2}$$

21 $$H{{O}_{2}}^{\bullet }+{{O}_{2}}^{\bullet -}\to H{{O}_{2}}^{\bullet -}+{O}_{2}$$

22 $$H{{O}_{2}}^{\bullet -}+{H}^{+}\to {H}_{2}{O}_{2}$$

23 $${H}_{2}{O}_{2}+{{O}_{2}}^{\bullet -}\to H{O}^{\bullet }+H{O}^{-}+{O}_{2}$$

24 $${H}_{2}{O}_{2}+{e}^{-}\to H{O}^{\bullet }+H{O}^{-}$$

25 $${H}_{2}{O}_{2}+h\nu \to 2H{O}^{\bullet }$$

26 $$MB-H+{h}^{+}\to MB-{H}^{\bullet +}\leftrightarrow M{B}^{\bullet }+{H}^{+}$$

27 $$MB-H+H{O}^{\bullet }\to M{B}^{\bullet }+{H}_{2}O$$

28 $$M{B}^{\bullet }+{O}_{2}\to MB-O{O}^{\bullet }\mathop{\to }\limits^{{H}_{2}O/{O}_{2}\,}Degradation$$

29 $$MB-H+H{O}^{\bullet }\to H-MB-H{O}^{\bullet }\mathop{\to }\limits^{{H}_{2}O/{O}_{2}\,}Degradation$$

The enhanced photocatalytic efficiency of the ZnO-Cu_x_O_3 core-shell nanowires as compared to the reference pristine ZnO and Cu_x_O layers is attributed to the contribution of ZnO-Cu_x_O staggered gap radial heterojunction to the separation and transport of the photogenerated charge carriers.

## Conclusions

A water stable photocatalytic heterostructure for degradation of organic pollutants (MB was used as a model dye) was obtained by covering thermally oxidized ZnO nanowires with an optimum thickness of Cu_x_O layer by magnetron sputtering, on one side for protecting ZnO nanowires from dissolution and on the other side for an improved charge transport towards the surface. The ZnO nanowires have a wurtzite crystalline structure, with the width of the band gap around 3.3 eV (n-type semiconductor), while the Cu_x_O is amorphous with a bang gap value about 1.74 eV (p-type semiconductor). The ZnO-Cu_x_O nanowires form a staggered gap radial heterojunction which enhances the separation and transport of the photogenerated charge carriers when irradiating with UV-light leading to MB degradation.

The proposed mechanism for the degradation of MB is described taking into consideration the dissolution of ZnO nanowires until reaching the optimum thickness of the Cu_x_O shell. The staggered gap radial heterojunctions obtained for the ZnO-Cu_x_O_3 nanowires have a better photocatalytic response (higher efficiency and higher degradation rate constant) than the pristine ZnO nanowires, being in the same time stable in water based solutions.

## Supplementary information

Supporting Information

## Data Availability

The datasets supporting the conclusions of the current study are presented in the manuscript and supporting information.
