# Supplementary material for: Core-shell nanowire arrays based on ZnO and CuxO for water stable photocatalysts
Source: Sci Rep. 2019 Nov 21;9:17268. doi: 10.1038/s41598-019-53873-0 (PMC6872873; doi:10.1038/s41598-019-53873-0)
Supplement: Supplementary file 1 — Supporting Information [file 41598_2019_53873_MOESM1_ESM.docx]

**Supporting Information**

Core-shell nanowire arrays based on ZnO and Cu_x_O for water stable photocatalysts

Camelia FLORICA,^¥†1^ Andreea COSTAS, ^¥†1^ Nicoleta PREDA,^1^ Mihaela BEREGOI,^1^ Andrei KUNCSER,^1^ Nicoleta APOSTOL,^1^ Cristina POPA,^2^ Gabriel SOCOL,^2^ Victor DICULESCU,^1^ Ionut ENCULESCU^†1^

^1^National Institute of Materials Physics, Multifunctional Materials and Structures Laboratory, Functional Nanostructures Group, 405A Atomistilor Street, 077125, Magurele, Ilfov, Romania

^2^National Institute for Laser, Plasma and Radiation Physics, 409 Atomistilor Street, 077125 Magurele, Ilfov, Romania

^¥^Camelia Florica and Andreea Costas contributed equally to this work.

^*^Corresponding author: [camelia.florica@infim.ro](mailto:camelia.florica@infim.ro) (C. Florica)

^**^Corresponding author: [andreea.costas@infim.ro](mailto:andreea.costas@infim.ro) (A. Costas)

^***^Corresponding author: [encu@infim.ro](mailto:encu@infim.ro) (I. Enculescu)


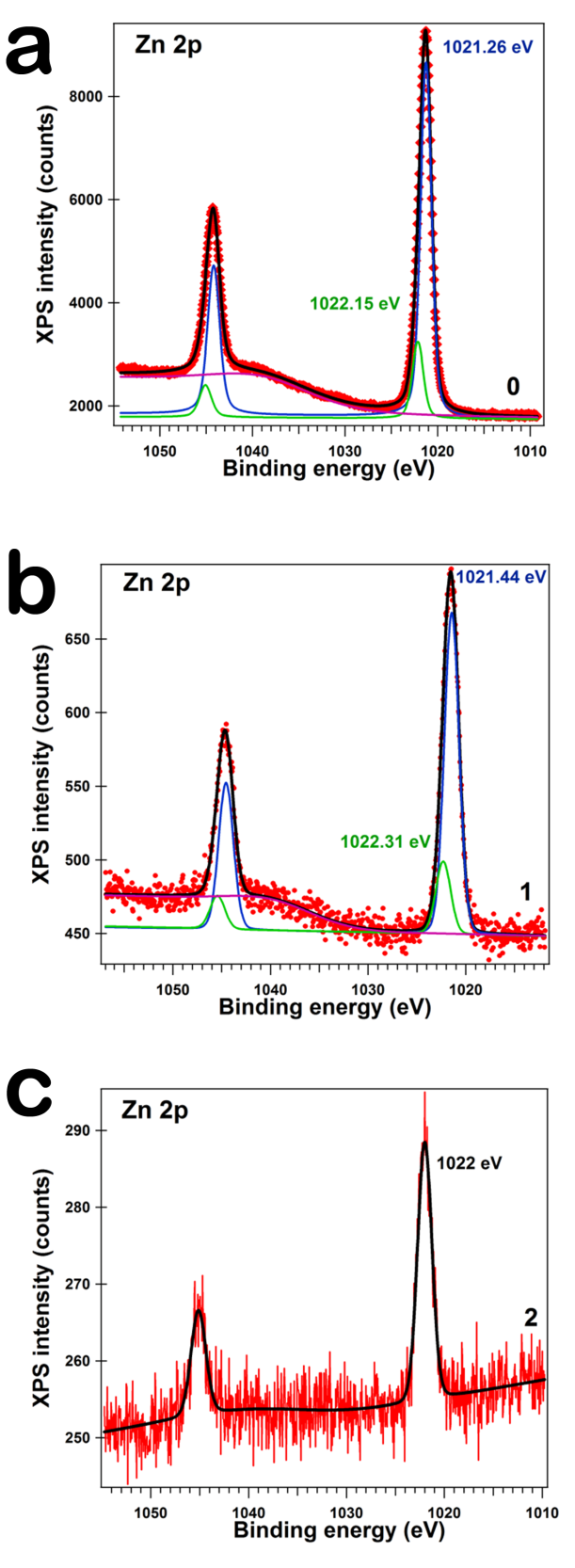


***Figure S1****. High resolution X-ray photoelectron spectroscopy of the Zn 2p levels for:*

*(a) ZnO, (b) ZnO-Cu_x_O_1, (c) ZnO-Cu_x_O_2*


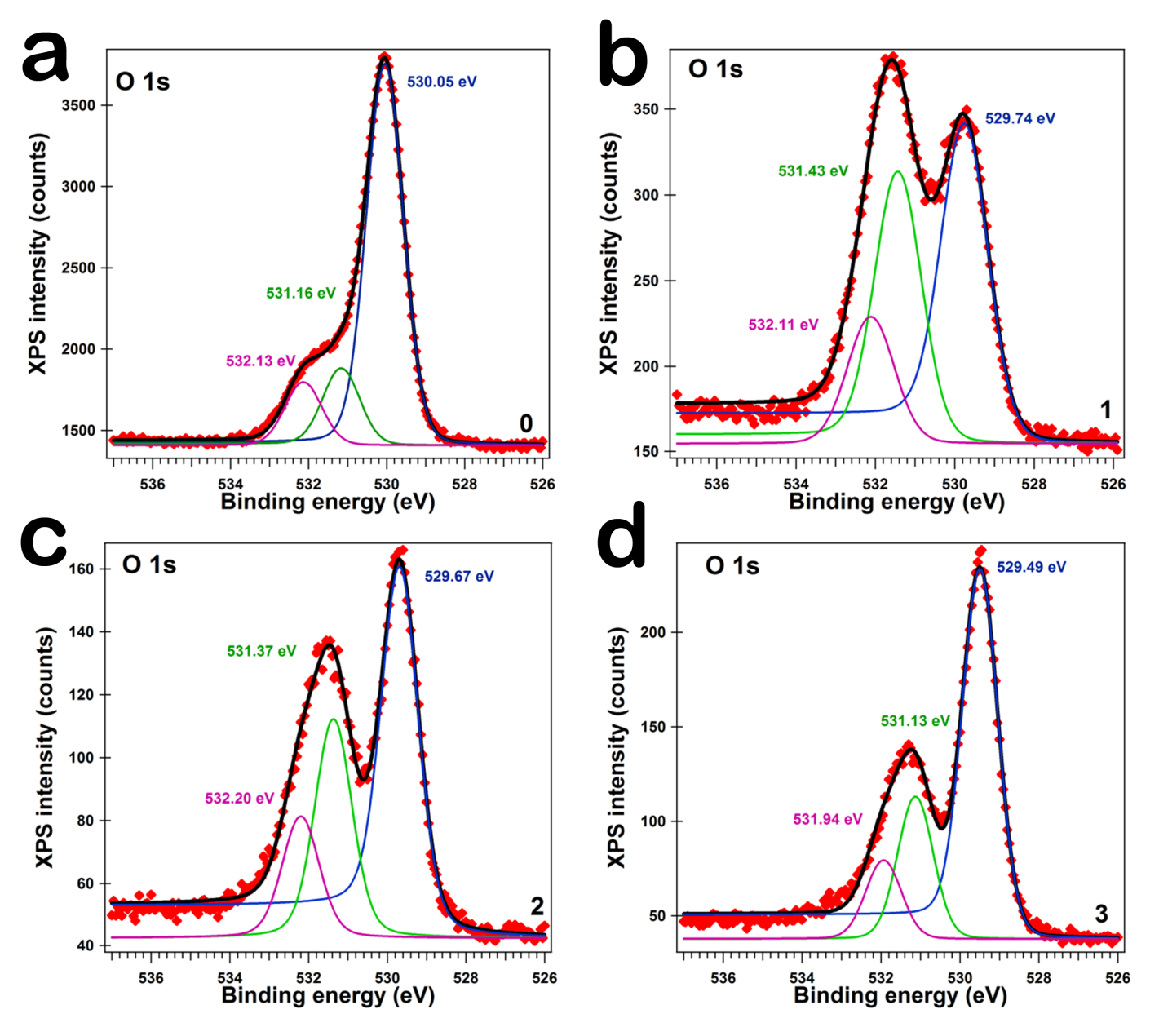


***Fi******gure S2****. High resolution X-ray photoelectron spectroscopy of the O 1s levels for: (a) ZnO,*

*(b) ZnO-Cu_x_O_1, (c) ZnO-Cu_x_O_2, (d) ZnO-Cu_x_O_3*


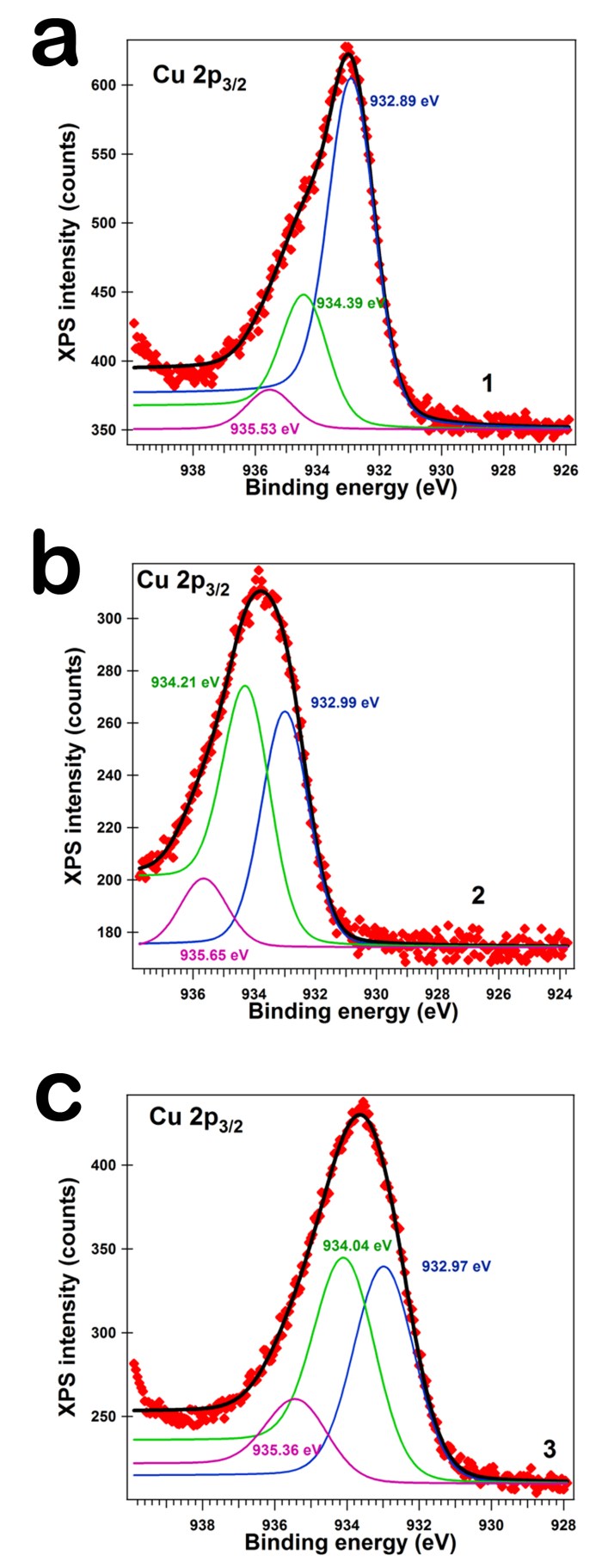


***Figure S3****. High resolution X-ray photoelectron spectroscopy of the Cu 2p_3/2_ levels for:*

*(a) ZnO-Cu_x_O_1, (b) ZnO-Cu_x_O_2, (c) ZnO-Cu_x_O_3*

***Table S1.*** *Ratios of the atomic compositions for all type of samples were determined using the integral areas provided by the deconvolution procedure normed to the atomic sensitivity factors*

| Sample | **Ratio**  **Cu(I)/Cu(II)** | **Ratio CuCO_3_/[Cu_2_O+CuO]** | **Ratio**  **ZnCO_3_/ZnO** |
| --- | --- | --- | --- |
| **ZnO** | - | - | 0.22 |
| **ZnO-Cu_x_O_1** | 2.72 | 0.09 | 0.23 |
| **ZnO-Cu_x_O_2** | 1.04 | 0.15 | 0.10 |
| **ZnO-Cu_x_O_3** | 1.05 | 0.18 | - |
